# Risk factors for neonatal sepsis among neonates in the neonatal intensive care unit at Hawassa University Comprehensive Specialized Hospital and Adare General Hospital in Hawassa City, Ethiopia

**DOI:** 10.3389/fped.2023.1092671

**Published:** 2023-04-17

**Authors:** Nigusie Shifera, Filagot Dejenie, Gebremeskel Mesafint, Tewodros Yosef

**Affiliations:** ^1^ School of Public Health, College of Medicine and Health Science, Mizan Tepi University, Mīzan Teferī, Ethiopia; ^2^ Public Health Emergency Management Department, Southwest Region Health Bureau, Tarcha, Ethiopia; ^3^ Psychiatry Department, College of Medicine and Health Science, Mizan Tepi University, Mīzan Teferī, Ethiopia

**Keywords:** neonatal sepsis, Hawassa city, neonate, case–control, Hawassa, Ethiopia

## Abstract

**Background:**

Neonatal sepsis (NS) is a serious blood bacterial infection in children of 28 days or younger, manifested by systemic signs and symptoms of infection. Neonatal sepsis has become one of the leading causes of admission and death in developing countries like Ethiopia. Understanding different risk factors for neonatal sepsis is essential for early diagnosis and treatment. So, this study aimed to assess the risk factors for neonatal sepsis among neonates at Hawassa University Comprehensive Specialized Hospital and Adare General Hospital in Hawassa City, Ethiopia.

**Methods and materials:**

A case–control study design was employed on 264 neonates (66 cases and 198 controls) in Hawassa University Comprehensive Specialized Hospital and Adare General Hospital from April to June 2018. Data were collected by interviewing the mothers and reviewing neonates’ medical records. The data were edited, cleaned, coded, and entered into Epi info version 7 and were transported and analyzed using SPSS version 20. The odds ratios (ORs) with 95% confidence intervals (CIs) were used to assess the significance of the associations.

**Result:**

A total of 264 neonates (66 cases and 198 controls) with 100% response rate. The mean (±SD) age of mothers was 26 ± 4.042 years. The majority (84.8%) of the cases were found in children under 7 days, with a mean age of 3.32 days ± 3.376 SD. Factors such as prolonged rupture of the membrane [AOR = 4.627; 95% CI (1.997–10.72)], history of the urinary tract or sexually transmitted infections [AOR = 2.5; 95% CI (1.151–5.726)], intrapartum fever [AOR = 3.481; 95% CI (1.18–10.21)], foul smelling liquor [AOR = 3.64; 95% CI (1.034–12.86)], and low APGAR score in the fifth minute [AOR = 3.38; 95% CI (1.107–10.31)] were the independent predictors of neonatal sepsis.

**Conclusion:**

Prolonged rupture of the membrane, intrapartum fever, urinary tract infection, foul-smelling liquor, and low APGAR score were independent risk factors of neonatal sepsis, and this study also observed that the onset of neonatal sepsis was higher in the first week of a neonate's life. Routine sepsis evaluation must focus on neonates born with the aforementioned characteristics and make interventions for babies born with these risk factors.

## Introduction

Neonatal sepsis (NS) is a serious life-threatening bacterial infection in neonates of 28 days or younger (1, 2). It is divided into early-onset neonatal sepsis (birth to 7 days, usually <72 h) and late-onset sepsis (from 8 to 28 days after birth) (3, 4). A culture and sensitivity test is a definitive diagnostic tool for neonatal sepsis. However, this gold-standard testing method is time-consuming (5). Usually, neonatal sepsis is diagnosed based on a combination of clinical findings and using positive septic screening parameters such as lymphocyte count, C-reactive protein, and erythrocyte sedimentation rate (6). A neonate with sepsis may require treatment aimed at the overwhelming systemic effects of the disease (7).

Globally, neonatal sepsis is one of the common causes of morbidity and mortality during the neonatal period (0–28 days). Out of 5.9 million child deaths in 2015, almost 1 million deaths occurred on the first day of life and approximately 2 million deaths occurred in the first week of life. Among the main cause of neonatal deaths, neonatal sepsis was the third cause, accounting for 15% of deaths globally (8). In developed countries, the incidence of neonatal sepsis varies from one to four cases per 1,000 live births, with significant differences over time and geography (3). Ninety-eight percent of global neonatal deaths occur in developing countries, especially in sub-Saharan Africa (9, 10).

According to the UNICEF child mortality report, one in 12 children in sub-Saharan Africa died before reaching their fifth birthday, which is much higher than the average ratio of one in 147 in high-income countries (11). Both maternal and neonatal factors play an important role in the early onset of sepsis (12, 13). In Ethiopia, the neonatal mortality rate (NMR) was reported to be 29/1,000 live births as of 2016 EDHS, which revealed a reduction from 37/1,000 live births in 2011. About 44% of neonatal deaths occur within the first 28 days of life, thus accounting for a larger proportion of under-five deaths. Out of all risk factors, neonatal sepsis accounts for 9% (14).

Despite improvements in the diagnosis and management, neonatal sepsis has become a leading cause of admission and death in neonatal units, especially in developing countries, due to sociodemographic, maternal, and neonatal conditions (15). Identification of bacteria and treatment is often unsatisfactory due to the nonspecific clinical presentation of sepsis and the lack of rapid diagnostic tests. Because sepsis diagnoses is difficult and time-consuming, the diagnostic approach should consider different risk factors for neonatal sepsis. Therefore, understanding the risk factors for NS could help in early diagnosis and treatment of the disease (16).

A limited amount of information on the risk factors for newborn sepsis in Africa and other developing nations was accessible despite the significant burden of neonatal sepsis (8). A delay in diagnosis of the disease could contribute to a delay in treatment, increasing the mortality rate by 50%. To prevent the delay in diagnosis, understanding common risk factors for neonatal sepsis is essential to help early diagnosis and treatment of the disease (17).

Studies have been conducted to determine the prevalence and risk factors for neonatal sepsis in neonates in both developing and developed countries (4, 9, 12, 13). In Ethiopia, limited studies pointed out the different factors for neonatal sepsis, namely, history of maternal UTI/STI (4, 18–20), place of delivery (4, 18), mode of delivery (18, 21), APGAR score <7/5 min (4, 19, 21), premature rupture of membrane (4, 19, 20), respiratory distress syndrome (19, 20, 22), intrapartum fever (4), birth weight (21), meconium aspiration (22), gestational week (20, 21), and not crying immediately at birth (4). However, no study has been performed in southern Ethiopia by incorporating more than one hospital. So, this study aimed to assess the risk factors of neonatal sepsis among neonates at Hawassa University Comprehensive Specialized Hospital and Adare General Hospital in Hawassa city

The result of this study will help policymakers and other responsible bodies to plan a necessary training program for health professionals, which helps them to improve their knowledge regarding the problem and identification of common risk factors. This could help to overcome the challenges of early diagnosis and management of sepsis. This will also help to provide valuable information to health professionals to create awareness in the community and give health education to mothers about various risk factors at the time of ANC follow-up and helps in early screening and treatment of the problem.

## Methods and materials

### Study area, design, and period

A facility-based unmatched case–control study was conducted at Hawassa University Comprehensive Specialized Hospital and Adare General Hospital of Hawassa from 15 April to 15 June 2018. Both hospitals were located in southern Ethiopia in Hawassa City, which is 268 km from Addis Ababa. The town had an estimated population of 401,177 in 2017, of whom 51.4% were females and 49.9% were males. Hawassa University Comprehensive Specialized Hospital serves about 18 million people from SNNPR and neighboring regions, over 90,200 outpatients, 18,116 hospitalized patients, and 1,092 emergency cases per year. Adare General Hospital provides health services to about 1.4 million people from the city and neighboring regions, over 100,000 outpatients, 5,104 hospitalized patients, and 3,743 emergency cases annually (23).

### Study population

All neonates with and without sepsis who were admitted and treated in the Hawassa University Comprehensive Specialized Hospital and Adare General Hospital and who fulfilled the eligibility criteria during the study period were included. Both hospitals follow the same guidelines for the diagnosis of neonatal sepsis.

Cases were neonates admitted to NICU with sepsis and aged less than or equal to 28 days at these two hospitals. Controls were neonates who did not fulfill the criteria for sepsis and who were admitted to the pediatric ward or neonatal ICU of both hospitals.

### Eligibility criteria

All neonates admitted and treated in the Hawassa University Comprehensive Specialized Hospital and Adare General Hospital were included; however, neonates with incomplete patient chart information and neonates with no index mothers were excluded.

### Operational definitions

*Neonatal sepsis:* neonates with sepsis within 0–28 days of life; *early-onset neonatal sepsis:* neonates with sepsis within 0–7 days of life; *late-onset neonatal sepsis:* neonates with sepsis within 8–28 days of life (24). The established IMNCI (Integrated Management of Neonatal and Childhood Illness) clinical features of neonatal sepsis along with hematological criteria were used to diagnose neonatal sepsis in both hospitals (25).

*Premature infant*: a live-born infant delivered before 37 weeks of pregnancy (based on the Ballard score or from the first day of the last menstrual period (26).

*Low birth weight*: a neonate whose birth weight is less than 2,500 g (27).

*Prolonged rupture of the membrane (PROM*): the time from membrane rupture to the onset of delivery is more than 18 h (28).

### Sample size determination and sampling technique

The sample size was calculated by the double population proportion formula using Epi Info version 7 by considering and assuming the case–control ratio (1:3), 95% confidence level, and 80% power. In a similar study, a male neonate was taken as the main predictor of neonatal sepsis. P1 represents the proportion of cases exposed to male neonates (22%), and P2 represents the proportion of controls exposed to male neonates (42.1%) (29). Then, after considering a 10% nonresponse rate, the total sample size was estimated to be 264 (66 cases and 198 controls).

Cases and controls were selected using a simple random sampling technique from the study population among neonates who attended both Hawassa University Comprehensive Specialized Hospital and Adare General Hospital during the study period. Before data collection, we skimmed some cases and are controls admitted and registered per month; then, we gave random numbers from previously recorded data of 88 cases and 239 controls of sampling frame picked by a lottery method for 66 cases and 198 controls admitted during the data collection period. Controls and cases were taken from the same hospital.

### Study variables

Neonatal sepsis (yes, no) was the dependent variable, and socio-demographic characteristics, neonatal risk factors, and maternal risk factors were the independent variables.

### Data collection and quality control

The data collection tool was prepared by reviewing a similar study (4, 18). Initially, the tool was prepared in English, translated to the local language (Sindamagna), and backtranslated to English to ensure consistency.

The data were collected by interviewing the mothers with their index neonates and reviewing neonatal medical records throughout the data collection period. A pretest was done among 5% of the study sample size, and training was provided to the data collectors and supervisors on the objective of the study, maintenance of ethical standards, and methods of data collection. Data completeness was checked, and data were cleaned and compiled by the investigator and a supervisor daily.

One supervisor and four data collectors with professional midwifery nurses/nurses participated based on their experience and skills. Data collectors were trained by the principal investigator on the objective of the study and procedures of data collection. The questionnaire was prepared in English, translated into Amharic, and then translated back into English to check for consistency. Also, data completeness was checked before entering the data into Epi-data 3.1. Cross-checking printout data for probable inaccuracies served as the basis for data coding and cleaning. Furthermore, descriptive analysis was used to check for missing values and outliers.

### Data processing and analysis

The data were edited, cleaned, coded, and entered into Epi info version 7 and exported to SPSS version 20 for analysis. The descriptive statistics were presented in frequencies, tables, and graphs. The normality assumption was checked for continuous variables. The candidate variables for multivariate logistic regression and bivariate logistic regression were used to investigate the relationship between neonatal sepsis and each variable. Multiple logistic regression analysis was used to find independent predictors of neonatal sepsis using variables with a *P*-value of less than 0.25. Multicollinearity between independent predictor variables was checked *via* tolerance and variance inflation factor methods. The Hosmer–Lemeshow test, which considers good fit at *P*-value >0.05, was used to verify the final model's goodness of fit. Finally, independent predictors of neonatal sepsis were identified using a 0.05 cutoff *P*-value, and the strength of the link was evaluated using AORs with their respective 95% confidence intervals.

### Ethical consideration

Ethical clearance was obtained from the Institutional Review Board of the College of Medicine and Health Sciences, Hawassa University. A support letter was obtained from the local authorities of both hospitals. Confidentiality was maintained by not exposing or sharing the information gathered from the respondents at all levels of the study. Mothers with their index neonates were selected for the study, and their verbal and written consent was obtained; those who refused to be interviewed were informed that they have the right to quit the study at any time. Permission paper was submitted to each responsible body ahead of data collection.

## Results

### Sociodemographic characteristics

A total of 264 neonates (66 cases and 198 controls) were included, providing a 100% response rate. The mean (±SD) age of mothers was 26 ± 4.042 years, ranging from 19 to 38 years. Regarding marital status, 59 (89.4%) respondents from cases and 174 (96%) controls were married. Thirty-three (50%) cases and 130 (49.7%) controls were housewives by occupation, and 8 (12.1%) cases and 23 (11.6%) controls did not attend formal education. The Orthodox religion was practiced by 88 (44.4%), followed by the Protestant religion by 77 (38.9%) among both cases’ mothers and controls’ mothers. The majority (56, 84.8%) of the cases and 174 (87.9.5%) controls were found under the age of 7 days, with a mean age of 3.32 days ± 3.376 SD (see Table 1).

**Table 1:** Socio-demographic characteristics of cases and controls attending Hawassa University Comprehensive Specialized Hospital and Adare General Hospital, Hawassa city, Ethiopia, 2018.

Variable	Case no (%)	Control no (%)	Total no (%)
**Maternal age**			
<20	6 (9.1)	18 (9.15)	24 (9.1)
21–34	5 (86.4)	167 (84.3)	224 (84.8)
>35	3 (13)	13 (6.6)	16 (6.1)
**Marital status**			
Single	4 (6.1)	13 (6.6)	17 (6.4)
Married	59 (89.4)	174 (87.9)	233 (88.3)
Divorced/separated	3 (4.5)	11 (5.6)	14 (5.3)
**Religion**			
Orthodox	28 (42.4)	88 (44.4)	116 (43.9)
Muslim	9 (13.6)	25 (12.6)	34 (12.9)
Catholic	3 (4.5)	8 (4.0)	11 (4.2)
Protestant	26 (39.4)	77 (38.9)	103 (39)
**Ethnicity**			
Sidama	27 (40.9)	82 (41.4)	109 (41.3)
Amhara	11 (16.7)	19 (9.6)	30 (11.4)
Wolayta	12 (18.2)	53 (26.8)	65 (24.6)
Oromo	9 (13.6)	22 (11.1)	31 (11.7)
Other	7 (10.6)	22 (11.1)	29 (11.0)
**Maternal education**			
No education	8 (12.1)	23 (11.6)	31 (11.7)
Primary	15 (22.7)	58 (29.3)	73 (27.7)
Secondary	24 (36.4)	65 (32.8)	89 (33.7)
College and higher	19 (28.8)	52 (26.3)	71 (26.9)
**Occupation**			
Housewife	33 (50.0)	97 (49.0)	130 (49.2)
Civil servant	11 (16.7)	28 (14.1)	39 (14.8)
Businesswomen	10 (15.2)	36 (18.2)	46 (17.4)
Private organization	3 (4.5)	10 (5.1)	13 (4.9)
**Age of neonate in days**			
≤7	56 (84.8)	174 (87.9)	230 (87.1)
>7	10 (15.2)	24 (12.1)	34 (12.9)
**Sex of neonate**			
Male	39 (59.1)	107 (54.0)	146 (55.3)
Female	27 (40.9)	91 (46.0)	118 (44.7)

### Maternal characteristics

The median (IQ range) of parity for both cases and controls was 2, ranging from 1 to 6 live births. All of the respondents from cases and controls attended antenatal care service (ANC) follow-ups at least once during their pregnancy. More than three-fourths of neonates (308, 80.4%) were delivered spontaneously, and 460 (82.6%) were delivered at the hospital. History of hypertension during pregnancy was found in 13 (6.6%) respondents from cases and 5 (7.6%) respondents from controls. The history of urinary tract infection during index pregnancy was 22 (33.3%) and 27 (13.6%) in respondents from cases and controls, respectively. Regarding intrapartum fever, 13 (19.7%) respondents from cases and 9 (4.5%) respondents from controls had a history of fever during labor. More than three-fourths of respondents from cases (21, 31.8%) and 17 (8.6%) respondents from controls had a history of prolonged rupture of the membrane (see Table 2).

**Table 2:** Maternal characteristics of cases and controls attending Hawassa University Comprehensive Specialized Hospital and Adare General Hospital, Hawassa city, Ethiopia, 2018.

Variable	Case *N* (%)	Control *N* (%)	Total *N* (%)
**Parity**			
Primi	17 (25.8)	49 (24.7)	66 (25.0)
Multi	49 (74.2)	149 (75.3)	198 (75)
**ANC**			
<3	9 (13.6)	27 (13.6)	36 (13.6)
≥3	57 (86.4)	171 (86.4)	228 (86.4)
**Place delivery**			
Hospital	60 (90.9)	184 (92.9)	244 (92.4)
Health center	6 (9.1)	14 (7.1)	20 (7.6)
**Mode of delivery**			
Spontaneous	42 (63.6)	42 (63.6)	42 (63.6)
Instrumental	7 (10.6)	7 (10.6)	7 (10.6)
Cesarean section	17 (25.8)	17 (25.8)	17 (25.8)
**Premature rupture of membrane**			
Yes	21 (31.8)	17 (8.6)	38 (14.4)
No	45 (68.2)	181 (91.4)	226 (85.6)
**Per vaginal examination**			
<3	42 (63.6)	138 (69.7)	180 (68.2)
>3	24 (36.4)	60 (30.3)	84 (31.8)
**Intrapartum fever**			
Yes	13 (19.7)	9 (4.5)	22 (8.3)
No	53 (80.3)	189 (95.5)	242 (91.7)
**Foul smelling liquor**			
Yes	6 (9.1)	8 (4.0)	14 (5.3)
No	60 (90.9)	19 (96.0)	250 (94.7)
**Hypertension during pregnancy**			
Yes	5 (7.6)	9 (4.5)	14 (5.3)
No	61 (92.4)	189 (95.5)	250 (94.7)
**Bleeding during pregnancy**			
Yes	6 (9.1)	15 (7.6)	21 (8.0)
No	60 (90.9)	183 (92.4)	243 (92.0)
**UTI/STI**			
Yes	22 (33.3)	27 (13.6)	49 (18.6)
No	44 (66.7)	171 (86.4)	215 (81.4)

### Neonatal characteristics

More than three-fourths (20, 30.3%) of cases and 27 (13.6%) controls had a gestation age of fewer than 37 weeks. Forty-two (56.1%) cases and 161 (81.3%) controls had a gestational age between 37 and 42 weeks of gestation. Post-term delivery have been recorded in 14 (5.1%) cases and 4 (6.1%) controls. Regarding the birth weight, 169 (85.4%) controls and 47 (71.2%) cases were delivered with normal birth weight. The mean gestational age and birth weight of both cases and controls were 38.4 ± 2.24 weeks and 2,999 ± 623 gr, respectively. The proportion of neonates who had an APGAR score <7 at the first minute was higher in the cases(24, 36.4%) than in controls (17, 8.6%). The proportion of neonates who had an APGAR score <7 at the fifth minute was also higher in cases (18, 27.3%) than in controls (7, 3.5%). Twenty-five (15.7%) controls and 21 (31.8%) cases were resuscitated at birth (see Table 3).

**Table 3:** Neonatal characteristics of cases and controls attending Hawassa University Comprehensive Specialized Hospital and Adare General Hospital, Hawassa city, Ethiopia, 2018.

Variable	Case no (%)	Control no (%)	Total no (%)
**Gestational age**			
<37 weeks	20 (30.3)	27 (13.6)	47 (17.8)
37–42 weeks	42 (63.6)	161 (81.3)	203 (76.9)
>42 weeks	4 (6.1)	10 (5.1)	14 (5.3)
**Birth weights**			
<2,500	19 (28.8)	29 (14.6)	48 (18.2)
≥2,500	47 (71.2)	169 (85.4)	216 (81.8)
**APGAR score at first minute**			
<7	24 (36.4)	17 (8.6)	41 (15.5)
≥7	42 (63.6)	181 (91.4)	223 (84.5)
**APGAR score at fifth minute**			
<7	18 (27.3)	9 (4.5)	27 (10.2)
≥7	48 (72.7)	189 (95.5)	237 (89.8)
**Resuscitation**			
Yes	21 (31.8)	25 (12.6)	46 (17.4)
No	45 (68.2)	173 (87.4)	218 (82.6)

### Determinants of neonatal sepsis

Cesarean section mode of delivery, UTI/STI during pregnancy, foul-smelling liquor, maternal fever, birth weight, APGAR score <7 in the first minute, APGAR score <7 in the fifth minute, and resuscitation at birth were subjected to the final model (multivariate) analysis to adjust for possible confounders, and these variables had *p*-value ≤ 0.25 in bivariable logistic regression analysis. In multivariable logistic regression analysis, maternal fever, UTI/STI during pregnancy, PROM, and APGAR score <7 in the fifth minute showed a significant association with neonatal sepsis.

Accordingly, PROM showed a significant association with the risk of neonatal sepsis. The odds of neonatal sepsis among mothers who gave birth after 18 h of rupture of membrane were 4.62 times higher than those among mothers who gave birth before 18 h of rupture of membrane [AOR = 4.627; 95% CI (1.997–10.72)]. Neonates born to mothers who had UTI/STI during the index pregnancy had 2.5 times higher odds of developing sepsis than those neonates who were born to mothers who did not have a UTI/STI during the index pregnancy [AOR = 2.5; 95% CI (1.151–5.726)]. Maternal fever had a significant association with the risk of neonatal sepsis; neonates born to mothers who had a fever during labor had 3.481 times higher odds of developing sepsis than those neonates whose mothers did not have a fever during labor [AOR = 3.481; 95% CI (1.18–10.21)]. Foul-smelling liquor was significantly associated with the risk of neonatal sepsis. Specifically, women with a history of foul-smelling liquor during labor were 3.64 times more likely to give neonates with neonatal sepsis than those without a history of foul-smelling liquor AOR = 3.64; 95% CI (1.034–12.86)].

Neonates who had APGAR score <7 in the fifth minute had higher odds of developing sepsis than neonates who had an APGAR score >7 [AOR = 3.38; 95% CI (1.107–10.31)] (see Table 4).

**Table 4:** Multivariable logistic regression analysis of risk factors of neonatal sepsis at Hawassa University Comprehensive Specialized Hospital and Adare General Hospital, Hawassa city, Ethiopia, 2018.

Variables	Case *N* (%)	Control *N* (%)	COR	AOR
**Mode of delivery**				
Spontaneous	42 (63.6)	154 (77.8)	1	1
Instrumental	7 (10.6)	13 (6.6)	1.97 (0.74–5.26)*	1.57 (0.48–5.04)
CS	17 (25.8)	31 (15.7)	2.01 (1.01–3.98)*	2.11 (0.91–4.86)
**Premature rupture of membrane**				
Yes	14 (21.2)	22 (11.1)	2.15 (1.03–4.50)*	4.63 (1.99–10.72)*
No	52 (88.9)	176 (88.)	1	1
**UTI/ST**I				
Yes	22 (33.3)	27 (13.6)	3.16 (1.64–6.08)*	2.57 (1.15–5.72)*
No	44 (66.7)	171 (86.)	1	1
**Foul smelling liquor**				
Yes	6 (9.1)	8 (4)	2.37 (0.79–7.12)*	3.64 (1.03–12.86)*
No	60 (90.9)	190 (96)		
**Intrapartum fever**				
Yes	13 (19.7)	9 (4.5)	5.15 (2.08–12.70)*	3.48 (1.18–10.21)*
No	53 (80.3)	189 (95.7)	1	1
**Birth weight (g**)				
<2,500	19 (28.8)	169 (85.4)	2.35 (1.21–4.57)*	2.20 (0.96–5.04)
>2,500	47 (71.2)	29 (14.6)	1	1
**Apgar score first minute**				
<7	24 (36.4)	17 (8.6)	6.08 (3.01–12.32)*	2.06 (0.80–5.04)
≥7	42 (63.6)	181 (91.4)	1	1
**Apgar score fifth minute**				
<7	18 (27.3)	9 (4.5)	7.87 (3.33–18.62)*	3.38 (1.10–10.31)*
≥7	48 (72.7)	189 (95.5)	1	1
**Neonates’ resuscitation**				
Yes	21 (31.8)	25 (12.6)	3.23 (1.65–6.28)*	2.23 (0.99–4.99)
No	45 (68.2)	173 (87.4)	1	1

* indicates *p* value < 0.05.

## Discussion

This study addressed the risk factors for neonatal sepsis. Factors such as PROM, maternal UTI/STI, intrapartum fever, foul-smelling liquor, and APGAR score of <7 in the fifth minute were the independent predictors of neonatal sepsis. The majority (84.8%) of cases were found in neonates younger than 7 days, which showed that more of the cases were early onset neonatal sepsis. This study finding was almost consistent with those conducted in North Gonder and Deberezeit, Ethiopia (18, 30).

Prolonged rupture of the membrane had a significant effect on the development of neonatal sepsis. Neonates born to mothers who had prolonged rupture of the membrane more than 18 h before delivery had about five times higher odds of developing sepsis than neonates born to their counterparts. This is comparable with the studies conducted in Mekele Hospital, Ethiopia (2016) and Soweto hospital (2012), South Africa, (4, 10). This is supported by the body of science that prolonged leakage and premature rupture of membranes are considered major risk factors for sepsis because of the danger of ascending infection. After membrane rupture, microorganisms from the vagina could ascend into the amniotic sac, predisposing the baby to infection *in utero* (23, 29).

In our study, a history of the urinary tract or sexually transmitted infection was found to be one of the risk factors contributing to neonatal sepsis during pregnancy. Neonates born to mothers who had UTI/STI during pregnancy had 2.6 times higher odds of developing sepsis than neonates born to mothers who did not have UTI/STI. This finding is also comparable and consistent with other similar studies conducted elsewhere (4, 18, 29). This could be due to untreated UTI/STI during third-trimester pregnancy and labor following the colonization of the birth canal by the infectious agent, and the baby is likely to ingest some of these bacteria as it is being delivered through the birth canal (18, 29).

Intrapartum fever was one of the risk factors contributing to the development of neonatal sepsis. Our study showed that neonates born to mothers who had a fever during labor had 3.42 times higher odds of developing sepsis than neonates born to mothers who did not have a fever during labor. Similar findings were also observed in earlier studies conducted in different parts of the world, in Ethiopia (2016) and Pakistan (2014) (4, 22, 31). Intrapartum fever is indicative of infections that are frequently transmitted to the baby *in utero* or during passage through the canal, which usually causes early-onset sepsis (4, 18).

Another risk factor for neonatal sepsis was foul-smelling liquor. Multivariate analysis indicated that neonates born to mothers who had foul-smelling liquor during labor were 3.64 times more likely to develop neonatal sepsis. This finding is also consistent with different studies conducted in Ghana (2014), Bangladesh (2011), and Nepal (2006) (23, 29, 32). Foul-smelling liquor is a reliable feature of chorioamnionitis emitting the smell due to breakdown products of bacterial metabolism, and the infection is easily transmitted to the fetus *in utero* (32).

We also found that the APGAR score <7 in the fifth minute had a 3.38 times risk of developing neonatal sepsis. Neonates who had APGAR scores <7in the fifth minute had higher odds of developing sepsis than neonates who had APGAR scores >7. This finding is comparable to earlier studies from Tanzania and Indonesia (17, 33). Perinatal hypoxia-ischemia can be caused by several factors. Neonates with low APGAR scores tend to have poor adaptation to extrauterine life due to the stress experienced during labor or the APGAR score reflects the infant’s changed condition in response to the resuscitation performed; therefore, neonates are more prone to infection (17, 33).

Place of delivery has a significant association with neonatal sepsis (4, 18), but in our study, it was not a significant factor. The possible reason could be the high proportion of hospital delivery and no home delivery. Similarly, the mode of delivery was not a significant factor and was inconsistent with other studies in Ethiopia. Cesarean section and instrumental delivery were also possible risk factors for neonatal sepsis in Deberezeyit and Gondar (18, 21). The possible explanation for this result could be the differences in sample size, study design used, and settings.

Prematurity and low birth weight are known common risk factors for neonatal sepsis (2, 9, 21). This study did not show associations with neonatal sepsis. Similar studies conducted in Deberzeyit and Mekele in Ethiopia also did not show a significant association with neonatal sepsis (18). This could be due to the difference in study design used, smaller sample size, health-care delivery systems, and awareness of the health professionals regarding the prevention strategy of sepsis.

## Conclusion and recommendation

According to this study, prolonged rupture of the membrane, intrapartum fever, maternal UTI/STI infection, foul-smelling liquor, and low APGAR score at the fifth minute were the independent factors associated with neonatal sepsis. This study has also observed that the onset of neonatal sepsis was higher in the first week of the neonatal life. Health care providers that work in the maternal and child health unit at Hawassa City health facilities should conduct health education of mothers about risk factors like prolonged rupture of the membrane, intrapartum fever, maternal UTI/STI infection, foul-smelling liquor, and low APGAR score at the fifth minute during ANC follow-ups, which will help them to be screened and treated early.

## Data Availability

The original contributions presented in the study are included in the article/Supplementary Material; further inquiries can be directed to the corresponding author.
